# A cross-sectional analysis of diagnosis and management of chronic obstructive pulmonary disease in people living with HIV: Opportunities for improvement

**DOI:** 10.1097/MD.0000000000027124

**Published:** 2021-09-17

**Authors:** Jerry S. Zifodya, Matthew Triplette, Shahida Shahrir, Engi F. Attia, Kathleen M. Akgun, Grant W. Soo Hoo, Maria C. Rodriguez-Barradas, Cherry Wongtrakool, Laurence Huang, Kristina Crothers

**Affiliations:** a Department of Medicine, Section of Pulmonary Diseases, Critical Care, and Environmental Medicine, Tulane University School of Medicine, New Orleans, LA; b Division of Pulmonary, Critical Care & Sleep Medicine, Department of Medicine, University of Washington, Seattle, WA; c Fred Hutchinson Cancer Research Center, Seattle, WA; d Department of Medicine, Section of Pulmonary, Critical Care and Sleep Medicine, Veterans Affairs Connecticut Healthcare System, West Haven, CT; e Yale University School of Medicine, New Haven, CT; f Department of Medicine, Pulmonary, Critical Care and Sleep Section, Veterans Affairs Greater Los Angeles Healthcare System, Los Angeles, CA; g Infectious Diseases Section, Michael E. DeBakey Veterans Administration Medical Center and Department of Medicine, Baylor College of Medicine, Houston, TX; h Department of Medicine, Atlanta Veterans Administration Medical Center & Emory University School of Medicine, Atlanta, GA; i Department of Medicine, Zuckerberg San Francisco General Hospital, University of California San Francisco, San Francisco, CA; j Veterans Administration Puget Sound Health Care System, Seattle, WA.

**Keywords:** bronchodilators, chronic obstructive pulmonary disease, diagnostic errors, HIV, spirometry

## Abstract

Chronic obstructive pulmonary disease (COPD) is common in people living with HIV (PLWH). We sought to evaluate the appropriateness of COPD diagnosis and management in PLWH, comparing results to HIV-uninfected persons.

We conducted a cross-sectional analysis of Veterans enrolled in the Examinations of HIV-Associated Lung Emphysema study, in which all participants underwent spirometry at enrollment and reported respiratory symptoms on self-completed surveys. Primary outcomes were misdiagnosis and under-diagnosis of COPD, and the frequency and appropriateness of inhaler prescriptions. Misdiagnosis was defined as having an International Classification of Diseases (ICD)-9 diagnosis of COPD without spirometric airflow limitation (post-bronchodilator forced expiratory volume in 1-second [FEV1]/Forced vital capacity [FVC] < 0.7). Under-diagnosis was defined as having spirometry-defined COPD without a prior ICD-9 diagnosis.

The analytic cohort included 183 PLWH and 152 HIV-uninfected participants. Of 25 PLWH with an ICD-9 diagnosis of COPD, 56% were misdiagnosed. Of 38 PLWH with spirometry-defined COPD, 71% were under-diagnosed. In PLWH under-diagnosed with COPD, 85% reported respiratory symptoms. Among PLWH with an ICD-9 COPD diagnosis as well as in those with spirometry-defined COPD, long-acting inhalers, particularly long-acting bronchodilators (both beta-agonists and muscarinic antagonists) were prescribed infrequently even in symptomatic individuals. Inhaled corticosteroids were the most frequently prescribed long-acting inhaler in PLWH (28%). Results were overall similar amongst the HIV-uninfected.

COPD was frequently misdiagnosed and under-diagnosed in PLWH, similar to uninfected-veterans. Among PLWH with COPD and a likely indication for therapy, long-acting inhalers were prescribed infrequently, particularly guideline-concordant, first-line long-acting bronchodilators. Although not a first-line controller therapy for COPD, inhaled corticosteroids were prescribed more often.

## 1 Introduction

Chronic obstructive pulmonary disease (COPD) prevalence is higher in people living with HIV (PLWH) compared with HIV-uninfected individuals.^[1–4]^ The greater COPD prevalence is explained only in part by the higher rates of smoking in PLWH,^[1,2,5–7]^ and may be related to increased susceptibility to respiratory infections, immunocompromised state, and chronic inflammation.^[8–11]^ Prior studies suggest high rates of both misdiagnosis and underdiagnosis of COPD in the general population, ranging from 60% to >90%.^[12,13]^ COPD is misdiagnosed when patients carry the clinical diagnosis, but spirometry does not confirm airflow limitation (defined as a ratio of the forced expiratory volume in one second [FEV1] over the forced vital capacity [FVC] of <0.70, or alternatively below the lower limit of normal (LLN) on post-bronchodilator spirometry).^[14,15]^ Individuals are under-diagnosed when they are not recognized clinically to have COPD, but risk factors (e.g., smoking) are present and spirometry is consistent with the disease.

Inaccurate diagnosis of COPD has clinical consequences. Misdiagnosis is potentially harmful as it may result in use of inappropriate therapies and their associated adverse effects and failure to investigate other causes of symptoms. Under-diagnosis can result in a lack of appropriate therapies for COPD and puts patients at risk of worsening respiratory symptoms, exacerbations, and affects health-related quality of life. Both misdiagnosis and under-diagnosis of COPD may be more common in PLWH as HIV providers have been shown to have less awareness of current smoking status in PLWH.^[16]^ Furthermore, multimorbidity, which is very common amongst PLWH, has been associated with a decreased likelihood of appropriate management of COPD.^[17,18]^ Despite the prevalence of COPD, there are limited studies evaluating treatment of COPD in PLWH, particularly in relation to the presence or absence of a clinical COPD diagnosis and spirometry results. We determined misdiagnosis and under-diagnosis of COPD in a multicenter cohort of PLWH and uninfected individuals and evaluated prescription patterns of inhalers for COPD.

## 2 Methods

We performed a cross-sectional analysis of the Examinations of HIV-Associated Lung Emphysema (EXHALE) study. EXHALE enrolled 361 veterans with and without HIV who were participating in the Veterans Aging Cohort Study (VACS)^[19]^ between 2009 and 2012 in a pulmonary substudy, excluding those with acute respiratory infection or prior lung disease other than COPD and asthma.^[20,21]^ This analysis excluded 11 participants who did not have complete spirometry and 15 with a diagnosis of asthma (International Classification of Diseases [ICD]-9: 493.xx) without concurrent diagnosis of COPD and <10 pack-years of smoking, leaving 335 participants in the analytic cohort. EXHALE was approved by the Institutional Review Boards at participating institutions. All participants provided written informed consent.

Smoking and symptom history were obtained from self-completed questionnaires.^[20,22,23]^ Comorbidities were defined using validated ICD-9 codes.^[24,25]^ Trained respiratory therapists in clinical pulmonary function laboratories at participating sites obtained pre- and post-bronchodilator spirometry in participants at enrollment. Results were reviewed first by site pulmonologists for quality and then sent to the coordinating center; those not meeting acceptability and reproducibility criteria^[26]^ were excluded.

Participants also underwent non-contrast, chest CT scans at full inspiration per standard protocol at study enrollment. A board certified, thoracic radiologist who was blinded to the HIV status of the participants interpreted images and scored the semi-quantitative severity of emphysema as previously described.^[27]^

Spirometry-defined COPD required a post-bronchodilator ratio of forced expiratory volume in one second (FEV1) over the forced vital capacity (FVC) of <0.7. In sensitivity analyses, spirometry-defined COPD was defined as FEV1/FVC ratio <LLN.^[28]^ An ICD-9 diagnosis of COPD was defined by codes consistent with COPD (491.xx, 492.xx, 496) prior to enrollment. COPD was considered misdiagnosed when patients had an ICD-9 diagnosis, but spirometry did not confirm airflow limitation, and considered under-diagnosed when participants did not have an ICD-9 code for COPD but had spirometric airflow limitation. We obtained prescriptions for any inhalers in the 12 months prior to enrollment from Veterans Health Administration (VHA) pharmacy data.

Our primary analyses used spirometry as the global initiative for chronic obstructive lung disease (GOLD) standard for physiologic diagnosis of COPD. Because emphysema is a subtype of COPD that is more common amongst PLWH and providers may have considered patients with emphysema as having COPD, in secondary analyses we explored how defining COPD as radiographic emphysema with or without airflow limitation on spirometry would impact accuracy of diagnosis and appropriateness of medication prescriptions. As in prior studies, we defined emphysema as having >10% emphysema on chest CT.^[20,21,27]^

Chi-squared and Wilcoxon-rank sum tests were used to compare baseline characteristics, COPD, and inhaler prescriptions in PLWH versus uninfected-veterans as appropriate. Results were considered significant at a 2-sided *P*-value of <.05. All analyses were performed using Stata version 14.0 (StataCorp LP, College Station, TX).

## 3 Results

Of the 183 PLWH and 152 HIV-uninfected participants, the majority were smokers (Table 1). Median age was similar, 56 years (interquartile range [IQR] 51–60) in PLWH and 53 years (IQR 49–59) in uninfected participants. PLWH were more likely to be men (98% vs 90%). Most participants were smokers: 84% of PLWH and 82% of those uninfected were current or former smokers with a median pack-year history of 27 (IQR 14–42) and 24 (IQR 11–38) pack-years, respectively. A total of 118 (65%) PLWH and 80 (53%) uninfected participants had self-reported symptoms of chronic cough or phlegm production, and 46% of PLWH and 40% of uninfected participants reported dyspnea (Medical Research Council [MRC] dyspnea scale ≥2).

**Table 1:** Demographics and baseline characteristics.

	PLWH (n = 183)	HIV-(n = 152)
Age, years median (IQR)	56 (51–60)	53 (49–59)
Race		
Black, n (%)	134 (73%)	103 (68%)
White, n (%)	42 (23%)	45 (30%)
Other, n (%)	7 (3.8%)	4 (2.6%)
Ethnicity, (% Hispanic)	27 (15%)	24 (16%)
Male sex (%)	180 (98%)	137 (90%)
BMI, median (IQR)	26 (24–29)	30 (26–34)
Smoking status^∗^		
Never smoker, n (%)	29 (16%)	28 (18%)
Former smoker, n (%)	39 (21%)	37 (24%)
Current smoker, n (%)	114 (63%)	87 (57%)
Pack years^†^ in current and former smokers, median (IQR)	27 (14–42)	24 (11–38)
Self-reported symptoms		
Chronic cough and/or phlegm, n (%)	118 (65%)	80 (53%)
MRC Dyspnea score ≥2, n (%)	73 (46%)	53 (40%)
Inhaler prescriptions^‡^		
Any inhalers, n (%)	37 (20%)	23 (15%)
Long-acting inhalers, n (%)	17 (9.3%)	10 (6.6%)
ICS, n (%)	14 (7.7%)	9 (5.9%)
LABA, n (%)	7 (3.8%)	5 (3.3%)
LAMA, n (%)	2 (1.1%)	1 (0.7%)
FEV1/FVC post BD <0.7, n (%)	38 (21%)	28 (18%)
GOLD stage^§^		
Stage 1 (FEV1 ≥ 80% predicted)	21/38 (55%)	10/28 (36%)
Stage 2 (50% ≤ FEV1 < 80% predicted)	13/38 (34%)	16/28 (57%)
Stage 3 (30% ≤ FEV1 < 50% predicted)	4/38 (11%)	2/28 (7%)
Bronchodilator reversibility	14 (38%)	10 (36%)
ICD-9 diagnoses		
ICD-9 COPD diagnosis on enrollment, n (%)	25 (14%)	23 (15%)
ICD-9 diagnoses of both asthma and COPD, n (%)	6 (3.3%)	8 (5.3%)
ICD-9 asthma diagnosis without diagnosis of COPD, n (%)	26 (15%)	10 (6.6%)

BD = bronchodilator, BMI = body mass index, COPD = chronic obstructive pulmonary disease, FEV1 = forced expiratory volume in 1 second, FVC = forced vital capacity, GOLD = global initiative for chronic obstructive lung disease 2006, HIV- = HIV-uninfected participants, ICD-9 = International Classification of Diseases Ninth Revision, ICS = inhaled corticosteroids, LABA = long acting beta-agonists, LAMA = long acting muscarinic antagonists, MRC = Medical Research Council, PLWH = people living with HIV.

∗ Smoking was defined as never (<100 cigarettes in lifetime), former (last cigarette >12 months ago), or current.

† Pack-years were calculated based on years of smoking and average number of cigarettes per day.

‡ Medication data provided is for any inhalers in the year before EXHALE enrollment. Medications were classified as either short-acting bronchodilators (beta agonists and/or muscarinic antagonists), or long-acting controller medications. This latter group consisted of long-acting beta agonists (LABA), long-acting muscarinic antagonists (LAMA), inhaled corticosteroids (ICS) or combination inhalers which were counted as exposure to each component medication.

§ We calculated percent-predicted values using reference equations from the Third National Health and Nutrition Examination Survey (NHANES).

Despite the high prevalence of smoking and the high proportion with respiratory symptoms, only 18% of PLWH and 17% of uninfected had spirometry prior to enrollment. Spirometry-defined COPD was identified in 66 participants: 38 (21%) PLWH and 28 (18%) HIV-uninfected participants (Table 1); 94% were current or former smokers with median pack-years of 40 (IQR 23.5–50). An ICD-9 COPD diagnosis was present in 48 participants: 25 (14%) PLWH and 23 (15%) uninfected; 88% were current or former smokers.

PLWH were frequently misdiagnosed or underdiagnosed with COPD, similar to uninfected participants. Of 25 PLWH with an ICD-9 COPD diagnosis, 14 (56%) were misdiagnosed. Of those with an ICD-9 code for COPD, less than half had prior spirometry in their VHA records, without significant difference by HIV status. Of 38 PLWH with spirometry-defined COPD, 27 (71%) were underdiagnosed. Of note, 23 (85%) of these 27 underdiagnosed individuals had chronic respiratory symptoms (a composite of chronic cough, phlegm production, and/or dyspnea defined as Medical Research Council dyspnea score ≥2). The proportions who had COPD misdiagnosis (56% of PLWH and 70% of uninfected, *P* = .40) and underdiagnosis (71% of PLWH and 75% of uninfected, *P* = .85) were similar by HIV.

In the entire cohort, 20% of PLWH and 15% of the HIV-uninfected were prescribed any inhalers in the year prior to enrollment (Table 1). Both PLWH and uninfected participants with self-reported respiratory symptoms were more likely to be on any inhaler compared with those without respiratory symptoms (27% vs 4.8%, *P* = .003 for symptomatic vs asymptomatic PLWH and 21% vs 4.2%, *P* = .007 for symptomatic vs asymptomatic uninfected). The most commonly prescribed inhalers were short-acting bronchodilators; long-acting inhalers were prescribed in 9.3% of PLWH and 6.6% of those uninfected with inhaled corticosteroids (ICS) being the most common inhaler. Among PLWH and HIV-uninfected participants misdiagnosed with COPD, 13% and 22%, respectively, were on potentially inappropriate long-acting inhalers.

**Figure 1:**
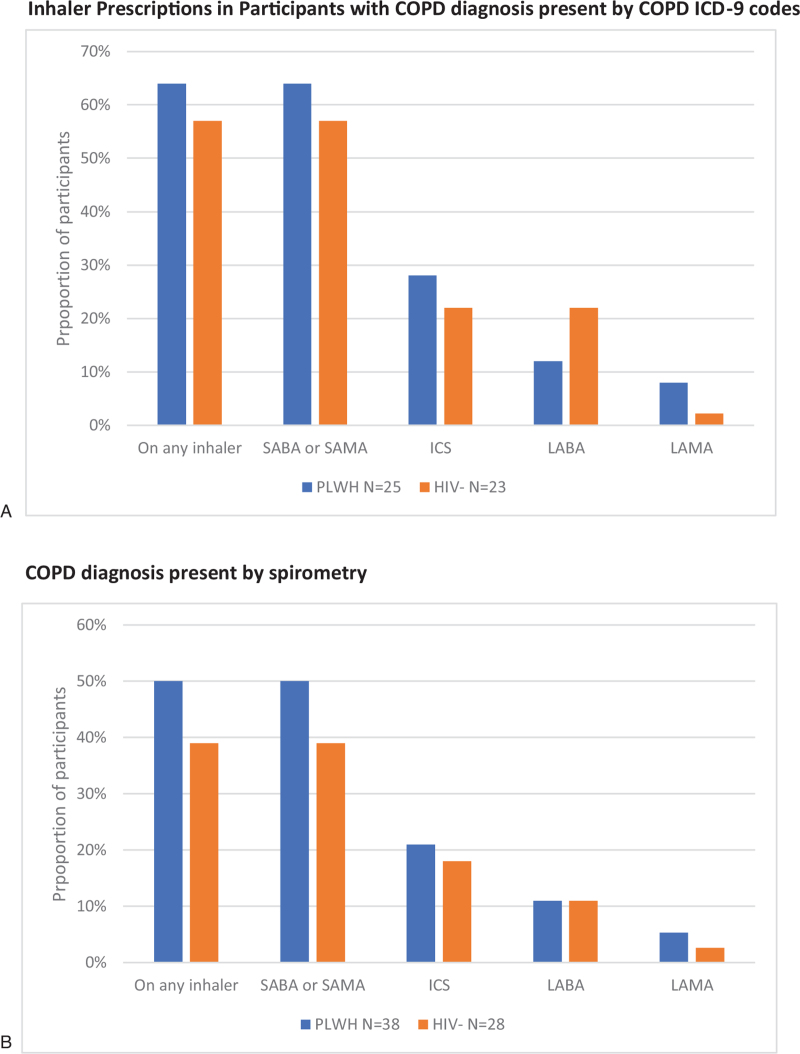
Comparison of inhaler use in participants with ICD-9 COPD and spirometric COPD 1-year pre-enrollment by HIV status^∗^. A. Inhaler prescriptions in participants with COPD diagnosis present by COPD ICD-9 codes. B. COPD diagnosis present by spirometry.^∗^ All comparisons by HIV-status were non-significant (*P* > .05). COPD = chronic obstructive pulmonary disease, HIV- = HIV-uninfected participants, ICD-9 = International Classification of Diseases Ninth Revision, ICS = inhaled corticosteroids, LABA = long-acting beta-agonists, LAMA = long-acting muscarinic antagonists, PLWH = people living with HIV, SABA = short acting beta-agonist, SAMA = short acting muscarinic antagonists.

Among participants with ICD-9 COPD diagnoses, long-acting inhalers were again prescribed infrequently with ICS being most common in both PLWH and uninfected (Fig. 1). Long-acting beta agonists (LABA) and long-acting muscarinic antagonists (LAMA) were prescribed less frequently in both groups, representing potential under-treatment, despite the high prevalence of chronic respiratory symptoms amongst patients with ICD-9 diagnoses of COPD (92% of PLWH and 93% of uninfected) and guideline recommendations at the time of this study for use of daily long-acting inhaler medications in addition to as-needed short-acting inhalers for symptomatic patients.^[14]^ Among participants with spirometry-defined COPD, results were similar.

In sensitivity analyses, 15 (23%) of the 66 participants with spirometry-defined COPD (FEV1/FVC < 0.7) had an FEV1/FVC ratio greater than the LLN; 7 (47%) were PLWH. Of these 15 participants, 3 (20%) had an ICD-9 diagnosis of COPD. There were no participants with an FEV1/FVC ration <LLN but >0.7.

A total of 108 of the 335 participants met criteria for COPD defined by spirometry showing airflow obstruction and/or radiographic emphysema. Similar to the main analyses, the proportion of participants who were misdiagnosed (42%) and under-diagnosed (73%) remained substantial. Notably, with this definition misdiagnosis was more common in PLWH (57%) as compared with HIV-negative participants (28%). The majority (76%) of those who were underdiagnosed had respiratory symptoms. Underdiagnosis was similarly common in PLWH (75%) as compared with HIV-negative participants (72%). The frequency of long-acting inhaler use, when including patients with emphysema, remained similarly low overall, with ICS the most frequently prescribed inhaler (data not otherwise shown).

## 4 Discussion

We found that COPD was both misdiagnosed and underdiagnosed frequently in PLWH. Few had spirometry for clinical purposes prior to study enrollment, highlighting the lack of appropriate work-up for chronic lung disease. Our findings of high rates of both misdiagnosis and under-diagnosis in PLWH are similar to those amongst HIV-uninfected participants in this study and those reported in the general, non-Veteran population.^[12,13]^ Findings were similar in sensitivity analyses in which we considered emphysema on chest CT to also signify COPD. However, defining spirometric COPD using the LLN did reduce the number of underdiagnosed participants. While other studies have reported on underdiagnosis of COPD in PLWH,^[29–31]^ this is one of the first to additionally report on misdiagnosis and to examine treatment of COPD in PLWH.

Further, we found that few participants were prescribed long-acting inhalers despite chronic respiratory symptoms, representing potential under-treatment. Of those individuals with spirometric COPD, 45% of PLWH and 64% of uninfected participants had GOLD stage ≥2, thus further demonstrating a potential indication for COPD therapy based on GOLD staging at the time.^[14]^ Additionally, because of misdiagnosis of COPD, inhalers were also often prescribed to those without spirometric airflow limitation. Of concern, the most commonly prescribed long-acting inhalers were ICS. While unclear if this was due to concomitant asthma, the lack of appropriate work-up (e.g., spirometry) and inaccurate diagnoses suggests that inhalers were also likely inappropriately prescribed in some individuals. Taken together, our results highlight the need for improvement in diagnosing chronic lung disease and quality of care for COPD amongst PLWH, as in HIV-uninfected patients. Because chronic respiratory symptoms of cough or phlegm production are more commonly reported in PLWH^[32–34]^ and symptoms are strongly associated with inhaler use, these findings underscore the need to pursue an accurate diagnosis of COPD in order to appropriately target therapy for COPD in PLWH.

LABA or LAMA are first-line long-acting inhalers for COPD per management guidelines^[14]^; however, as aforementioned, we found that ICS were the most commonly used long-acting inhalers. This is concerning given the increased risk for pneumonia associated with the use of ICS in COPD^[35,36]^ along with the concomitant increased risk for opportunistic pneumonia associated with underlying HIV. Further, ICS use is of concern in PLWH given the potential for medication interactions.^[37]^ ICS use is appropriate in participants who have asthma. While it is possible that participants were placed on ICS as therapy for asthma, we excluded those who were likely to have asthma alone from our main analyses by excluding participants with a diagnosis of asthma, no diagnosis of COPD, and <10 pack years of smoking. We cannot exclude that ICS prescriptions were appropriately given to individuals with asthma or asthma-COPD overlap (ACO). In addition, few of the participants were on triple therapy with LABA, LAMA, and ICS as is recommended for patients with frequent exacerbations of COPD. Taken together, our results raise the possibility that ICS use was inappropriate, and not fully explained by the presence of asthma, ACO, or frequent exacerbations of COPD. Given the increased risk of opportunistic pneumonia in PLWH, inappropriate ICS use could be even more harmful in this population as compared with the general population, and reinforces the need to improve uptake of evidence-based care of COPD amongst PLWH.

Our study has several strengths. First, the VHA electronic health record allows for a comprehensive investigation with very few missing data elements. Second, we were able to assess treatment in the context of participant self-reported symptoms. Third, we had robust measurements on participants, including pack-years of smoking and spirometry. In addition, we were able to evaluate whether including radiographic emphysema, which may be a more common manifestation of COPD in PLWH, changed our results in terms of appropriate diagnosis and treatment as we had research chest CT scans in participants; although more participants met criteria for COPD with this approach, results were otherwise similar.

Our study also had certain limitations. First, the sample with COPD was small. Second, inhaler use was defined by at least one prescription, and may represent an over-estimation of longer-term use. Third, our analyses rely on VHA prescriptions and may have missed prescriptions outside VHA. However, the majority of Veterans Aging Cohort Study participants receive their care within VHA without difference by HIV status,^[19]^ and Veterans who fill any medication prescriptions through VHA are more likely to fill all of them at VHA.^[38]^ Fourth, we excluded from our main analyses people more likely to have asthma without COPD; however, we cannot distinguish whether providers believed they were treating asthma alone, COPD, or ACO. Thus, it is unclear how many prescriptions for ICS were truly inappropriate. Finally, the study may not be generalizable as it consisted of mostly male Veterans with a high prevalence of smoking recruited specifically for a pulmonary study; however, the cohort was racially and geographically diverse, and compared demographically similar PLWH and uninfected in the same health care system.

## 5 Conclusion

In summary, we found that COPD was both frequently misdiagnosed and under-diagnosed to a similar extent in PLWH and uninfected Veterans. Inhalers were more likely to be prescribed for individuals with respiratory symptoms, many of whom did not have a confirmed spirometric diagnosis of COPD. However, long-acting inhalers were used infrequently to manage COPD (defined either by spirometry or ICD-9 codes) even in symptomatic individuals. Future studies are required to implement strategies to facilitate improved diagnosis and management of COPD in PLWH.

## Acknowledgments

The Department of Veterans Affairs did not have a role in the conduct of the study, in the collection, management, analysis, interpretation of data, or in the preparation of the manuscript. The views expressed in this article are those of the authors and do not necessarily represent the views of the Department of Veterans Affairs or the U.S. Government.

## Author contributions

All authors meet the four authorship criteria recommended by the ICMJE. Conceived and designed the analysis (Jerry Simbarashe Zifodya, Matthew Triplette, Shahida Shahrir, Kristina Crothers); contributed data or analysis tools (Jerry Simbarashe Zifodya, Matthew Triplette, Shahida Shahrir, Engi F. Attia, Kathleen M. Akgun, Grant W. Soo Hoo, Maria C. Rodriguez-Barradas, Cherry Wongtrakool, Laurence Huang, Kristina Crothers); performed the analysis (Matthew Triplette); drafted the manuscript (Jerry Simbarashe Zifodya, Matthew Triplette, Kristina Crothers).

**Conceptualization:** Jerry Simbarashe Zifodya, Matthew Triplette, Shahida Shahrir, Kristina Crothers.

**Data curation:** Jerry Simbarashe Zifodya, Matthew Triplette, Shahida Shahrir, Engi F. Attia, Laurence Huang, Kristina Crothers.

**Formal analysis:** Matthew Triplette, Shahida Shahrir.

**Funding acquisition:** Laurence Huang, Kristina Crothers.

**Investigation:** Jerry Simbarashe Zifodya, Matthew Triplette, Shahida Shahrir, Engi F. Attia, Kathleen M. Akgun, Grant W. Soo Hoo, Maria C. Rodriguez-Barradas, Cherry Wongtrakool, Laurence Huang, Kristina Crothers.

**Methodology:** Jerry Simbarashe Zifodya, Matthew Triplette, Shahida Shahrir, Engi F. Attia, Kathleen M. Akgun, Grant W. Soo Hoo, Maria C. Rodriguez-Barradas, Cherry Wongtrakool, Laurence Huang, Kristina Crothers.

**Project administration:** Kristina Crothers.

**Resources:** Laurence Huang, Kristina Crothers.

**Supervision:** Laurence Huang, Kristina Crothers.

**Writing – original draft:** Jerry Simbarashe Zifodya, Matthew Triplette, Kristina Crothers.

**Writing – review & editing:** Jerry Simbarashe Zifodya, Matthew Triplette, Shahida Shahrir, Engi F. Attia, Kathleen M. Akgun, Grant W. Soo Hoo, Maria C. Rodriguez-Barradas, Cherry Wongtrakool, Laurence Huang, Kristina Crothers.
